# Erythrocytes lacking the Langereis blood group protein ABCB6 are resistant to the malaria parasite *Plasmodium falciparum*

**DOI:** 10.1038/s42003-018-0046-2

**Published:** 2018-05-03

**Authors:** Elizabeth S. Egan, Michael P. Weekes, Usheer Kanjee, Jale Manzo, Ashwin Srinivasan, Christine Lomas-Francis, Connie Westhoff, Junko Takahashi, Mitsunobu Tanaka, Seishi Watanabe, Carlo Brugnara, Steven P. Gygi, Yoshihiko Tani, Manoj T. Duraisingh

**Affiliations:** 1 Department of Immunology and Infectious Diseases, Harvard T.H. Chan School of Public Health, Boston, 02115 MA USA; 2 Boston Children’s Hospital, Boston, 02115 MA USA; 3 Department of Pediatrics, Stanford University School of Medicine, Stanford, 93205 CA USA; 4 Department of Cell Biology, Harvard Medical School, Boston, 02115 MA USA; 5 The Laboratory of Immunohematology and Genomics, New York Blood Center, New York, 10065 NY USA; 6 Japanese Red Cross Kinki Block Blood Center, Osaka, Japan; 7 Japanese Red Cross Kyushu Block Blood Center, Fukuoka, Japan; 8 Department of Laboratory Medicine, Boston Children’s Hospital, Boston, 02115 MA USA; 9 Present Address: Cambridge Institute for Medical Research, University of Cambridge, Cambridge, CB2 0QQ UK

## Abstract

The ATP-binding cassette transporter *ABCB6* was recently discovered to encode the Langereis (Lan) blood group antigen. Lan null individuals are asymptomatic, and the function of ABCB6 in mature erythrocytes is not understood. Here, we assessed ABCB6 as a host factor for *Plasmodium falciparum* malaria parasites during erythrocyte invasion. We show that Lan null erythrocytes are highly resistant to invasion by *P. falciparum*, in a strain-transcendent manner. Although both Lan null and Jr(a-) erythrocytes harbor excess porphyrin, only Lan null erythrocytes exhibit a *P. falciparum* invasion defect. Further, the zoonotic parasite *P. knowlesi* invades Lan null and control cells with similar efficiency, suggesting that ABCB6 may mediate *P. falciparum* invasion through species-specific molecular interactions. Using tandem mass tag-based proteomics, we find that the only consistent difference in membrane proteins between Lan null and control cells is absence of ABCB6. Our results demonstrate that a newly identified naturally occurring blood group variant is associated with resistance to *Plasmodium falciparum*.

## Abstract (web summary)

Elizabeth Egan and colleagues demonstrate that host ATP binding cassette transporter ABCB6, which encodes the Langereis blood group antigen, promotes erythrocyte invasion by the malaria parasite *Plasmodium falciparum*. This study suggests that asymptomatic Langereis null individuals may be better protected from malaria.

## Introduction

Severe malaria is caused by the protozoan parasite *Plasmodium falciparum*. Endemic to much of the tropical developing world, malaria remains a leading cause of mortality among young children and pregnant women. *P. falciparum* invades erythrocytes during the blood stage of its life cycle when clinical illness occurs. Invasion involves a series of ordered molecular interactions between ligands expressed on the parasite surface and receptors on the erythrocyte plasma membrane. To date, all of the known receptors for *P. falciparum* encode polymorphic blood group proteins, including glycophorin A, complement receptor I, and Basigin, though the molecular identities of several putative receptors are unknown^1–3^.

The high-incidence Langereis (Lan) blood group antigen was recently found to be encoded by *ABCB6*^4^. ABCB6 is a member of the large ATP-binding cassette transporter family of proteins and has been shown to transport porphyrin in nucleated cells, but its physiologic role in enucleated erythrocytes is unknown^5–7^. Lan-negative individuals with null mutations in *ABCB6* are asymptomatic and have no evidence of anemia^4^. These observations suggest that ABCB6 does not have an essential function in porphyrin transport or heme biosynthesis in erythrocytes under normal physiologic conditions. However, a recent study of porphyria patients identified mutations in *ABCB6* as genetic modifiers associated with severe symptoms, suggesting that ABCB6 may play an export role during porphyrin overload^8^. Consistent with these findings, *ABCB6*-null mice are hematologically normal at baseline, but suffer increased mortality after a porphyrin-inducing stress^9^.

Missense mutations in *ABCB6* have been linked to various rare genetic disorders, including ocular coloboma, dyschromatosis universalis hereditaria (DUH), and familial pseudohyperkalemia (FP)^10–14^. Erythrocytes from patients with FP leak potassium ions during prolonged storage, but this phenotype has only been observed with dominant gain-of-function mutations, and is not a characteristic of Lan null erythrocytes^13^.

We recently performed a forward genetic screen of human blood group genes to identify host factors required for *P. falciparum* invasion of red blood cells derived ex-vivo from hematopoietic stem/progenitor cells (HSPCs)^15^. *ABCB6* was among the top ten hits, though the significance of hits at this level is unknown. Due to the localization of ABCB6 at the erythrocyte plasma membrane and the precedent that all known *P. falciparum* receptors encode blood group proteins, we hypothesized that it may help mediate parasite entry.

Here, we investigate this hypothesis by studying *P. falciparum* invasion into erythrocytes from Lan-negative and Lan-positive individuals. We demonstrate that *P. falciparum* depends on ABCB6 to establish a productive invasion event. This phenotype is observed for all *P. falciparum* strains tested and is independent of the specific *ABCB6*-null genotype. We find that parasite attachment to Lan null erythrocytes is impaired relative to control cells, confirming a phenotype during host cell invasion. Using quantitative mass spectrometry, we demonstrate that the unifying feature of the *P. falciparum*-resistant Lan null erythrocytes is the absence of plasma membrane ABCB6. Together, our findings indicate that ABCB6 is a critical host factor mediating *P. falciparum* invasion of human erythrocytes.

## Results

### Lan null erythrocytes are resistant to invasion by *P. falciparum*

**Fig. 1:**
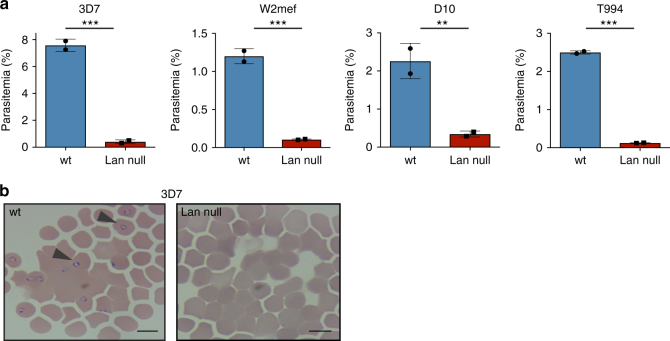
*Plasmodium falciparum* invasion is impaired in erythrocytes that lack the Lan antigen. Comparison of *P. falciparum* invasion into erythrocytes from a Lan null or normal donor. **a** Four different laboratory-adapted strains of *P. falciparum* (3D7, W2mef, D10, and T994) were incubated at the late schizont stage with wt or Lan null erythrocytes, and reinvasion was measured by SYBR green I staining and flow cytometry. Values are expressed as mean parasitemia ±SD, *n* = 2. ***p < 0.005, **p < 0.03, two-tailed *t*-test. **b** Images of *P. falciparum* invasion assays in control or Lan null erythrocytes. Arrows indicate ring-stage parasites. ×100 magnification. Scale bars are 10µm

*ABCB6* was recently identified as the locus encoding the Lan blood group, for which natural polymorphisms and nulls have been identified^4^. To further investigate the role of ABCB6 in host cell invasion by *P. falciparum*, we obtained a sample of Lan null primary human erythrocytes from the New York Blood Center (Table 1)^16^. We used this sample in invasion assays alongside a control, Lan-positive sample that had been donated and cryopreserved at a similar time. In these experiments, mature, schizont-stage *P. falciparum* parasites were allowed to invade the Lan null or Lan-positive erythrocytes, and newly infected cells with ring-stage parasites were quantified using flow cytometry. Laboratory-adapted *P. falciparum* strain 3D7 invaded control Lan-positive cells efficiently, while invasion into the Lan null cells was reduced by 95% (Fig. 1a). The same trend was observed for three additional laboratory-adapted *P. falciparum* strains. On thin blood smears, ring-stage parasites were visible within several hours in the Lan-positive cells, but were rarely seen in Lan-negative cells (Fig. 1b). These results suggest that Lan/ABCB6 plays a critical role during *P. falciparum* invasion of primary human erythrocytes.

**Table 1:** Lan null and control samples used in this study

Sample number	Phenotype	Identifying number	ABO	Ethnicity	Source	Cryopreserved?	ABCB6 nucleotide change	Putative amino acid change	Reference
Lan-NYBC	Lan null	VB IH2 B3C3	O	C	NYBC	Yes	c. 1558_1559insT homo	p.Val520CysfsStop	^16^
1	Lan null	L-3	O	J	JRC	Yes	20 A > G, 403 C > A hetero, 459delC hetero	p.Tyr7Cys, p.Arg135Ser, p.Leu154SerfsX97	^17^
2	Lan null	L-4	A	J	JRC	Yes	459delC homo	p.Leu154SerfsX97	^17^
3	Lan null	L-7	A	J	JRC	Yes	IVS16 + 1 g > a homo	splicing defect	^17^
4	Lan null	L-17	A	J	JRC	Yes	N.D.	N.D.	
5	Lan null	L-6	B	J	JRC	Yes	459delC hetero 1617delG hetero	p.Leu154SerfsX97, p.Gly539HisfsX15 hetero	^17^
6	Control (JMH-)	JMH-	B	J	JRC	Yes	N.D.	N.D.	
7	Control (Ko)	Ko	O	J	JRC	Yes	N.D.	N.D.	
8	Control Jk(a-b-)	Jk(a-b-)	A	J	JRC	Yes	N.D.	N.D.	
9	Control Jk(a-b-)	Jk(a-b-)	O	J	JRC	Yes	N.D.	N.D.	
10	Control	wt	O	J	JRC	No	N.D.	N.D.	
12	Control	wt	A	J	JRC	No	N.D.	N.D.	
13	Control	wt	B	J	JRC	No	N.D.	N.D.	
JR-1	Jr (a-)	JR-1	O	J	JRC	No	N.D.	N.D.	
JR-2	Jr (a-)	JR-2	O	J	JRC	No	N.D.	N.D.	

*N.D.* indicates not done, *ABO* ABO blood type, *C* Caucasian, *J* Japanese, *NYBC* New York Blood Center, *JRC* Japanese Red Cross

### Lan null cells with distinct genotypes are resistant to *P. falciparum*

**Fig. 2:**
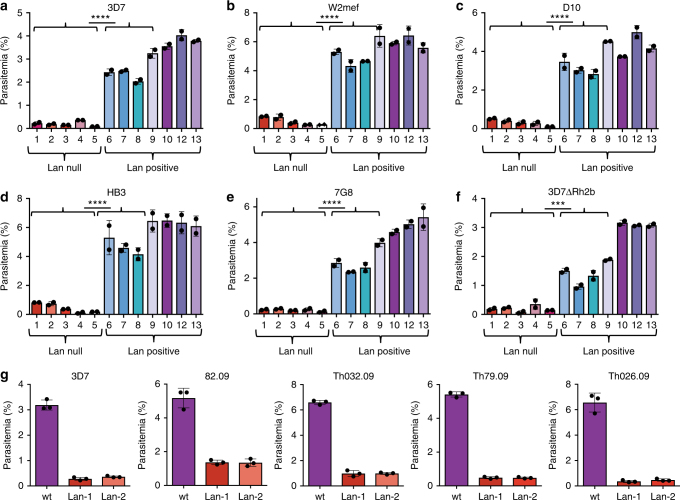
Impaired *P. falciparum* invasion into Lan-negative erythrocytes is independent of Lan null genotype, and is strain-transcendent. **a** Comparison of invasion by *P. falciparum* strain 3D7 into erythrocytes from five unrelated Lan null individuals (#1–5; see Table 1 for details of samples) and seven Lan-positive control individuals (#6–10 and 12–13), which serve as biological replicates. Samples 1–9 were previously cryopreserved and thawed, and samples 10,12, and13 were fresh. **b**–**f** Invasion by five additional *P. falciparum* strains (W2mef, D10, HB3, 7G8, and 3D7ΔRh2B, respectively). Invasion was measured by SYBR green I staining and flow cytometry. Values are expressed as mean parasitemia ±SD, *n* = 2. ****p ≤ 0.0001, ***p = 0.0002, two-tailed *t*-tests. **g** Invasion by *P. falciparum* strain 3D7 or four clinical isolates from malaria patients in Senegal (82.09. Th032.09, Th79.09, and Th026.09) into Lan null or Lan-positive erythrocytes. Invasion was measured by SYBR green I staining and flow cytometry. Values are expressed as mean parasitemia ±SD, *n* = 2

To further assess whether the invasion phenotype observed in the Lan null cells was attributable to the absence of Lan/ABCB6, we obtained erythrocyte samples from five additional Lan-negative donors with distinct null mutations in *ABCB6*, as well as seven Lan-positive donors, from the Red Cross Kinki Block Blood Center in Japan (Table 1)^17^. All of the Lan null and four of the Lan-positive samples had been cryopreserved, and the three additional Lan-positive samples were included as fresh controls. We used the cells in invasion assays with *P. falciparum* strain 3D7 and measured the formation of ring-stage parasites by flow cytometry. The results show that invasion into all of the Lan null samples was reduced by >90% compared to Lan-positive cells (Fig. 2a). These findings demonstrate that Lan null erythrocytes with different null mutations in *ABCB6* are all refractory to invasion by *P. falciparum* 3D7, and provide additional evidence for the specificity of ABCB6 as a required host factor for *P. falciparum* parasites.

### Lan null invasion phenotype is parasite strain-transcendent

During invasion, *P. falciparum* deploys specialized ligands localized in apical organelles to interact with receptors on the erythrocyte plasma membrane. Several of these interactions are believed to be redundant; different *P. falciparum* strains use distinct invasion pathways depending on which of the invasion ligands they express^18^. Current evidence suggests that only two host molecules, Basigin and CD55 (DAF), play essential roles during invasion for all *P. falciparum* strains^15,19^. To further assess whether the invasion phenotype in Lan null erythrocytes is strain-transcendent, we studied invasion by several additional laboratory-adapted strains in addition to 3D7 that preferentially use different ligand-receptor interactions (D10, W2mef, 7G8, HB3, and 3D7ΔRh2B^20^). We also used field isolates that had been adapted to culture directly from patients in Senegal (kindly provided by D. Ndiaye, S. Mboup, and S. Volkman). We observed that all of the diverse *P. falciparum* strains tested had impaired invasion into all of the Lan null erythrocyte samples, similar to strain 3D7 (Fig. 2b–g). These findings are consistent with a model where ABCB6 plays a critical, conserved, and strain-transcendent role in parasite invasion that is not influenced by strain-dependent differential expression of known invasion ligands.

### Impaired attachment to Lan null erythrocytes

**Fig. 3:**
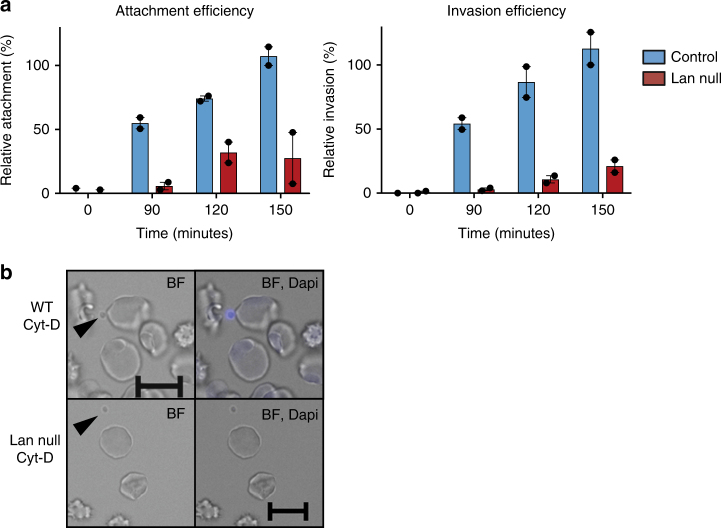
Attachment of *P. falciparum* to erythrocytes is dependent on ABCB6. **a** Mature *P. falciparum* strain 3D7 schizonts were added to normal wt (blue) or Lan null (red) erythrocytes in the presence of 1 μM cytochalasin-D to measure attachment or in the absence of cyt-D to measure invasion. Attachment and invasion were measured by SYBR green I staining and flow cytometry. Measurements were obtained every 30 min to account for progressive rupture of schizonts, and normalized for attachment/invasion measured in the presence of heparin, *n* = 2. Data represent the average of two biological replicates ±S.E.M. Values are expressed as relative to the attachment or invasion measured for a control sample at *T* = 150 min. **b** Representative images from attachment assay in the presence of Cyt-D showing wt or Lan null erythrocytes and *P. falciparum* merozoites (arrows). Scale bars = 10 μm

To further define the role of ABCB6/Lan in *P. falciparum* invasion of erythrocytes, we assessed the ability of parasites to attach to Lan null or Lan-positive erythrocytes using an attachment assay. *P. falciparum* schizonts were incubated with recipient erythrocytes in the presence of cytochalasin-D, which inhibits actin polymerization and parasite internalization, and allows the normally-transient attached state to be isolated and quantified^15,21^. Within 90 min after adding schizonts, we could readily detect daughter parasites (merozoites) attached to the Lan-positive cells, but the efficiency of attachment to the Lan-negative erythrocytes was reduced by more than 75% (Fig. 3a, b). These results mirrored the defect in invasion seen for the Lan null cells, confirming a role for ABCB6 early in the invasion process and not a later step in the parasite asexual cell cycle.

### Lan null invasion phenotype is not due to elevated porphyrin

**Fig. 4:**
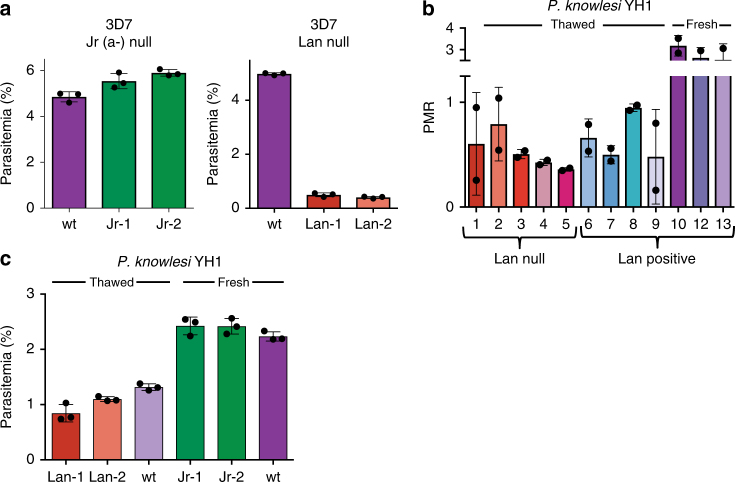
Impaired *P. falciparum* invasion into Lan null cells is a specific phenotype attributable to absence of ABCB6. **a** Invasion of *P. falciparum* strain 3D7 into Junior(a)- erythrocytes or Lan null erythrocytes, both of which have elevated intracellular porphyrin, compared to wt control cells. Values are expressed as mean parasitemia ±SD, *n* = 3. **b** Invasion by humanized *P. knowlesi* strain H1 into Lan null erythrocytes with distinct genotypes (#1–5) versus wt cells (#6–13). Values are expressed as mean parasite multiplication rate (PMR) over one cycle ±SD, *n* = 3. **c** Invasion by *P. knowlesi* YH1 into Lan null or Jr(a-) erythrocytes with distinct genotypes versus wt cells. Lan null and corresponding control cells were previously cryopreserved, while Jr(a-) cells and controls were freshly drawn. Values are expressed as mean parasitemia ±SD, *n* = 3

Although the physiologic role of ABCB6 is incompletely understood, several studies have implicated it in the transport of porphyrins, which are intermediates in the heme biosynthesis pathway^6,9,22^. Lan null erythrocytes have mildly increased intracellular porphyrin^4^, raising the possibility that the *P. falciparum* invasion phenotype we observed could be explained by porphyrin toxicity. To test this hypothesis, we used erythrocytes from two individuals with null mutations in the established porphyrin exporter *ABCG2*, which encodes the Jr^a^ blood group antigen^23,24^. Like Lan null erythrocytes, Jr(a-) erythrocytes have mildly elevated levels of intracellular porphyrin (1.2-fold and 1.7-fold increased over control cells, respectively)^24^. In contrast to the findings for Lan null cells, *P. falciparum* invaded the Jr(a-) erythrocytes normally, with an efficiency that was indistinguishable from wt erythrocytes (Fig. 4a). These results demonstrate that mild elevation of intracellular porphyrin does not inherently alter erythrocyte susceptibility to *P. falciparum* infection, and indicate that the invasion phenotype observed for the Lan null cells is not due to elevated porphyrin.

### Normal invasion of Lan null erythrocytes by *Plasmodium knowlesi*

To assess whether Lan null erythrocytes are inherently resistant to parasitism, we examined invasion by a humanized strain of the zoonotic parasite *Plasmodium knowlesi*. In contrast to *P. falciparum*, *P. knowlesi* invaded Lan-negative and Lan-positive erythrocytes with similar efficiency, suggesting that *P. knowlesi* invasion does not depend on ABCB6 (Fig. 4b). Similarly, *P. knowlesi* invaded Jr(a-) cells as well as control cells, suggesting that in both cell types invasion is unaffected by altered porphyrin levels (Fig. 4c). Although neither ABCB6 nor ABCG2 influenced *P. knowlesi* invasion, these parasites showed a clear preference for fresh as opposed to cryopreserved cells, reflecting their known tendency to invade younger erythrocytes^25^. Taken together, these results demonstrate that Lan null cells are not inherently resistant to infection by *Plasmodium* parasites, and suggest that *P. falciparum* may have a unique reliance on ABCB6 as a host factor during invasion.

### Plasma membrane profiling reveals specific loss of *ABCB6*

**Fig. 5:**
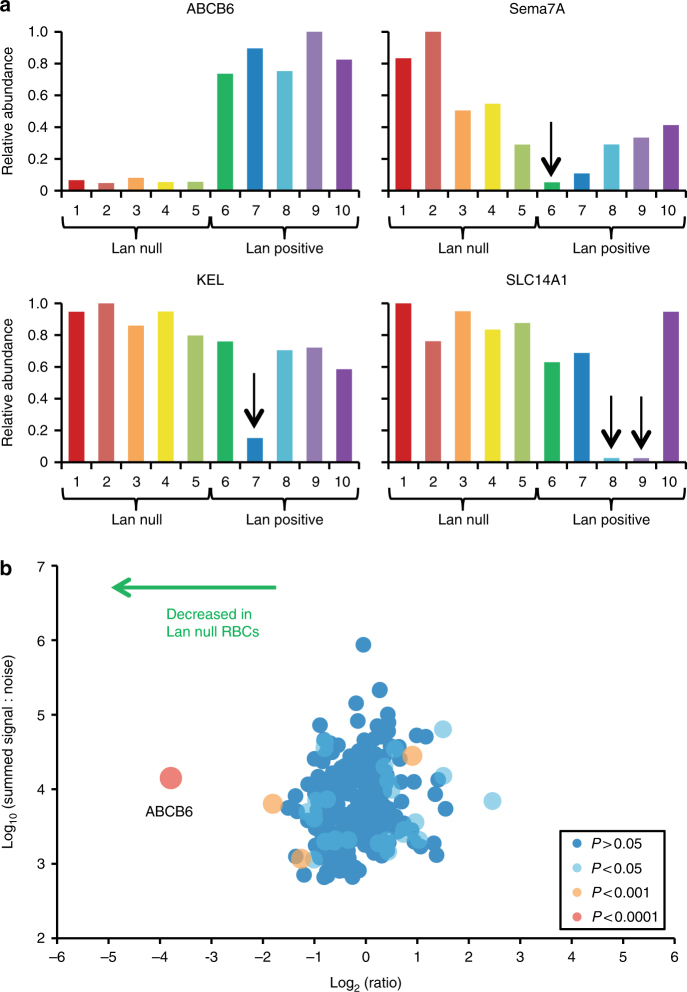
Plasma membrane profiling of Lan null and control erythrocytes reveals specific absence of ABCB6. **a** Quantification of ABCB6, Sema7A, KEL, and SLC14A1 proteins (which encode the Lan, JMH, Kell, and Kidd blood group antigens, respectively) in erythrocyte samples from 10 donors by tandem mass tag-based proteomic plasma membrane profiling. Donors 1–5 were known to be Lan null, and donors 6–10 known Lan-positive controls. Donor 6 was also known to be JMH null, donor 7 Kell null, and donors 8 and 9 were Kidd null. **b** Scatter plot of all 265 proteins quantified by two or more peptides and annotated ‘plasma membrane’ by Gene Ontology. The *x*-axis shows average fold change, calculated for each protein by sum (signal:noise) Lan null samples/sum (signal:noise) control samples. *P*-values were estimated using a two-tailed *t*-test, corrected for multiple hypothesis testing using the method of Benjamini–Hochberg

Our observation that Lan null erythrocytes from six unrelated donors are resistant to invasion by *P. falciparum* is consistent with a model where ABCB6 functions as a critical host factor during invasion. To determine whether other genetic differences and/or changes in protein expression between the Lan-positive and Lan-negative erythrocytes could explain or confound the results, we performed tandem mass tag-based proteomic plasma membrane profiling to compare the abundance of plasma membrane proteins between five Lan null and five Lan-positive samples^15,26^. We confirmed that ABCB6 was present at similar levels on all control cells, but present only at the level of noise on the plasma membranes of the five Lan null samples (Fig. 5a). Of the control samples, four were known to be negative for the JMH, Kell, or Kidd blood groups, and the proteomic analysis verified absence of the corresponding proteins (Sema7A, KEL, or SLC14A1), demonstrating that the technique worked as expected (Fig. 5a). By comparing the average abundance of each plasma membrane protein in the group of Lan null erythrocytes to control cells, we found that ABCB6 was the only significantly underrepresented protein common to the Lan null cells (*p* < 0.0001), indicating that absence of ABCB6 expression is not associated with loss in surface presentation of another plasma membrane protein (Fig. 5b and Supplementary Table 1). These findings indicate that the specific absence of ABCB6 underlies the invasion defect seen for the Lan null cells and support its role as a required host factor for *P. falciparum* invasion into human erythrocytes.

### Genetic variation in *ABCB6* in malaria-exposed populations

To determine whether there is evidence for natural selection for the Lan null blood type due to malaria, we analyzed human genetic data from the Human Exome Aggregation Consortium (ExAC), which includes sequencing data on more than 60,000 individuals^27^. We identified 52 coding or splice variants in *ABCB6* with a significantly different prevalence in human populations with high versus low exposure to malaria (*p* < 0.05; Supplementary Table 2). While 33 of these variants were more common in populations with high exposure to malaria, 19 were more common in populations with low malaria exposure. Only four of the identified 52 variants have previously been associated with the Lan null blood type, two of which were enriched in the populations with high malaria exposure, one at high prevalence (Table 2). The limited data available on the associations between *ABCB6* genotype and Lan blood type, particularly in African populations, limits conclusions as to whether *ABCB6* is under selection.

**Table 2:** The five most prevalent *ABCB6* coding variants enriched in high malaria exposure populations as percent of population, from the ExAC database

ΔAA^a^		p.Thr521Ser	p.Arg648Gln^d^	p.Leu425Val	p.Val306Ile	p.Ala511Thr
rs^b^		rs149363094	rs13402964	rs111852229	N.A.	rs140089441
Predicted effect		missense	missense/null	missense	missense	missense
Pops^c^	Exp^e^					
Afr	high	1.54	0.64	0.44	0	0.16
S.A.	high	0.12	0	0.01	0.26	0.01
E.A.	low	0	0.01	0	0	0
Fin	low	0.05	0	0	0	0
Euro	low	0.38	0.01	0	0	0
Lat	low	0.28	0.05	0.03	0	0
*P*-value^f^		7.63E-14	**1.31E-30**	**2.44E-26**	**3.73E-27**	**7.67E-10**

^a^ Amino acid change and location

^b^ Reference SNP cluster ID (NCBI)

^c^ Populations:*Afr* Africans, *S.A.* South Asians, *E.A.* East Asians, *Fin* Finnish, *Euro* non-Finnish Europeans, *Lat* Latinos

^d^ Variant previously reported for Lan null blood type

^e^ Exposure of current or ancestral popluation to malaria based on World Health Organization DALY

^f^ Fisher’s exact test comparing populations binned for high or low malaria exposure

## Discussion

Here we demonstrate that erythrocytes from Lan null individuals lacking plasma membrane protein ABCB6 are resistant to invasion by *P. falciparum* parasites. Using Lan-negative cells from six unrelated donors with distinct null genotypes, we showed that parasite invasion was impaired in the absence of ABCB6 in all cases. Similar results were observed for diverse laboratory-adapted and clinical isolates of *P. falciparum*, suggesting that the resistance phenotype is not restricted to parasite strains from a particular geographic region or expressing a specific dominant invasion ligand. These findings indicate that the reliance of *P. falciparum* on ABCB6 is strain-transcendent, similar to the strain-transcendent requirements for Basigin and CD55 during invasion^3,15,19^.

Results from attachment assays showed that *P. falciparum* displayed impaired attachment to the Lan null cells as compared to Lan-positive cells, further supporting a role for ABCB6 during parasite invasion. Entry of *P. falciparum* into erythrocytes is a fast, multi-step process involving a series of ordered interactions. The initial encounter involves parasite merozoite surface proteins, which mediate a reversible, weak deformation of the erythrocyte surface^2,28^. Next, the parasite reorients apically and strongly deforms the erythrocyte surface, leading to irreversible attachment. This step requires interactions between strain-specific erythrocyte binding-like and reticulocyte binding-like parasite ligands and alternative receptors on the erythrocyte plasma membrane that are believed to be functionally redundant, including glycophorins A, B, and Complement Receptor 1 (CR1). Although our studies point to a role for ABCB6 during attachment, they support a model in which its function is distinct from many of the known ligand-receptor interactions that occur during apical reorientation that are believed to be strain-dependent interactions with alternative receptors. As we found that all *P. falciparum* strains tested rely on ABCB6 for erythrocyte invasion, our findings support a model where ABCB6 plays a conserved role during invasion, similar to that of Basigin, perhaps by binding to a parasite ligand expressed by all *P. falciparum* strains or by serving a critical function independent of ligand-receptor interactions, such as establishment of the tight junction. Additional experiments will be necessary to determine exactly when and how ABCB6 functions relative to the other events known to occur during invasion.

Our data demonstrating a defect in parasite attachment to Lan null cells suggests that the role of ABCB6 in *P. falciparum* invasion is independent of porphyrin export. ABCB6 has been shown to transport protoporphyrin IX (PPIX) and other disease-related porphyrins in mouse reticulocytes; its PPIX transport function is redundant with ABCG2^8^. Likely due to this redundancy, absence of ABCB6 appears to only be physiologically meaningful in the setting of clinical porphyria in both mice and humans, consistent with Lan null individuals being asymptomatic^4,9^. We demonstrated that the mildly elevated porphyrin concentrations found in Lan null cells could not account for the observed invasion phenotype, because *P. falciparum* invaded Jr(a-) erythrocytes normally even though they also have elevated porphyrin. Consistent with these findings, it has been shown that parasites grow normally in porphyrin-laden erythrocytes from patients with X-linked dominant protoporphyria, even though PPIX accumulates >40-fold above normal levels in these cells^29^. The finding that ABCB6 in mouse cells can transport porphyrins that ABCG2 does not raises the caveat that this may also occur in human cells^8^.

An alternative explanation for the impaired invasion we observed could be a general membrane defect making Lan null RBCs refractory to parasitism, rather than a specific requirement for ABCB6. However, evidence from our studies suggests this is not the case. We showed that the zoonotic *Plasmodium* parasite *P. knowlesi* invaded Lan null and Lan-positive erythrocytes equally, demonstrating that Lan null cells are not inherently resistant to *Plasmodium* spp. invasion. In support of this interpretation, Lan null erythrocytes are structurally normal in terms of cell size and hemoglobin concentration, and neither anemia nor dyserythropoiesis have been reported in Lan null individuals^4^. The conclusions of our study are further bolstered by quantitative mass spectrometry data characterizing the plasma membrane proteome of the Lan null and Lan-positive erythrocytes. These experiments demonstrated that ABCB6 was the only plasma membrane protein universally depleted in all of the Lan null samples, minimizing the possibility that another protein could account for the observed phenotypes. In particular, none of the erythrocyte membrane proteins with established roles in *P. falciparum* invasion varied between the Lan null and control samples, including CD44, CD55, Basigin, complement receptor I, and the glycophorins. However, this method of plasma membrane profiling does not evaluate the internal cellular milieu or organization of proteins at the surface, so we cannot fully rule out the possibility that the requirement for ABCB6 is indirect.

The Lan antigen is considered a high-incidence blood group antigen^30^. Since the recent identification of *ABCB6* as the genetic locus encoding Lan, several null alleles have been described, some of which appear to be specific to different ethnic populations^4,16,17,31,32^. In our studies of *P. falciparum*, we found an invasion defect phenotype that was common to Lan null erythrocytes with distinct null mutations in ABCB6. These results raise the intriguing possibility that the Lan null blood type may provide resistance to malaria in clinical settings. Such an association between a blood type variant and full resistance to invasion has never been described for *P. falciparum*, but would be analogous to the situation for *P. vivax* and the Duffy antigen, where individuals that are Duffy-negative are resistant to *vivax* malaria^33^.

Our analysis of *ABCB6* human genomic data indicated that many coding variants are differentially enriched in populations with high versus low ancestral malaria exposure. However, since the prevalence of the Lan null phenotype has primarily been assessed in European and East Asian populations, future studies focused on populations with a high incidence of endemic malaria will be required to assess genotype–phenotype correlations for *ABCB6*/Lan and address the question of whether *ABCB6* is under selection due to malaria. The Lan null phenotype has only been reported in two individuals with African ancestry^34^. Ultimately, genetic association studies will be necessary to determine if there is an epidemiologic link between the Lan null blood type and protection from clinical malaria. The findings from our in vitro studies suggest *P. falciparum* has a unique reliance on ABCB6 as a host factor during invasion. As Lan null individuals are believed to be asymptomatic, this suggests that ABCB6 may have potential as a target for a new, host-directed therapy for malaria.

## Methods

### Parasite culture

The *P. falciparum* strains 3D7, W2Mef, HB3, D10, 7G8, and T994 are standard laboratory-adapted strains that originate from distinct geographic regions^35–37^ and were obtained from MR4 or the Walter and Eliza Hall Institute (Melbourne, Australia). Field strains were isolated from patients in Senegal, adapted to short-term culture and were provided by D. Ndiaye, S. Mboup, and S. Volkman^15^. *Plasmodium knowlesi* YH1 is a human-adapted strain that has been previously described^25^. *Plasmodium spp*. were routinely cultured in human O^+^ erythrocytes at 2% hematocrit in RPMI-1640 (Sigma) supplemented with 25 mM HEPES, 50 mg L^–1^ hypoxanthine, 2.42 mM sodium bicarbonate, and 4.31 mg ml^–1^ Albumax (Invitrogen) at 37 °C in 1% O_2_, 5% CO_2_. Field strains were cultured in inactivated human AB^+^ serum.

### Human erythrocyte samples

All Lan null erythrocytes were cryopreserved specimens, and corresponding control erythrocytes included both cryopreserved and fresh cells. Cryopreserved cells were thawed before use using standard methods and stored initially in Alsever’s solution and then in RPMI-1640 medium (Sigma) supplemented with 25 mM HEPES and 50 mg L^–1^ hypoxanthine and used within 2 weeks. Jr(a-) erythrocytes and corresponding fresh controls were freshly drawn and used within 2 weeks.

### Invasion assays

For invasion assays using erythrocytes from Japanese Red Cross, schizont-stage parasites were isolated using a MACS magnet (Miltenyi) and added at 1% parasitemia to acceptor erythrocytes that had been washed and resuspended in complete RPMI at 0.5% hematocrit in a total of 100μl per well in 96-well plates. For invasion assays using erythrocytes from NYBC, acceptor erythrocytes were at 0.2% hematocrit in 100 μl per well and *P. falciparum* schizonts were added at 0.5% (w2mef), 1.5% (D10), 1.5% (T994), or 3.8% (3D7) initial parasitemia.

Assays were performed in duplicate at 37 °C in 1% O2 and in 5% CO2. After overnight incubation, the cells were fixed in 4% paraformaldehyde and 0.0075% glutaraldehyde, stained with 1:2000 SYBR Green I, and run on a MACSQuant to quantify parasitemia. Flow cytometry data were analyzed with FlowJo software (V.10.0.8).

### Attachment assays

Attachment assays were performed as described previously with few modifications^15^. Acceptor erythrocytes were resuspended at 0.5% hematocrit in complete RPMI in a 12-well plate in a volume of 1000 μl per well. Assays were performed in duplicate. Synchronized *P. falciparum* strain 3D7 schizonts were isolated using a MACS magnet, and added to acceptor erythrocytes at 15% initial parasitemia in the presence or absence of 1 μM cytochalasin-D, to allow attachment, but to prevent parasite entry. Wells with 50 U ml^–1^ heparin, which prevents early attachment, were used as a negative control. Aliquots were taken at timepoints of 0 min, 90 min, 120 min, 150 min, and fixed in 0.116 M sucrose and 2% glutaraldehyde, and stained in 1:1000 SYBR Green I for 20 min, and analyzed for DNA content on a Cytek DxP flow cytometer. Data were analyzed using FlowJo software (V.10.0.8).

### Erythrocyte plasma membrane profiling

Plasma membrane profiling was performed as previously described^15,38^. Briefly, 1 × 10^8^ of each erythrocyte sample were washed with PBS. Surface sialic acid residues were oxidized with sodium meta-periodate (Thermo) then biotinylated with aminooxy-biotin (Biotium). After quenching, cells were incubated in 1% Triton X-100 lysis buffer. Biotinylated glycoproteins were enriched with high affinity streptavidin agarose beads (Pierce) and washed extensively. Captured protein was denatured with DTT, alkylated with iodoacetamide (IAA, Sigma), and digested on-bead with trypsin (Promega) in 200 mM HEPES pH 8.5 for 3 h. Tryptic peptides were collected and labeled using TMT reagents. The reaction was quenched with hydroxylamine, and TMT-labeled samples combined in a 1:1:1:1:1:1:1:1:1:1 ratio. Labeled peptides were enriched, desalted, and separated into six fractions using tip-based strong cation exchange. Mass spectrometry data was acquired and searched as previously described using an Orbitrap Fusion coupled with a Proxeon EASY-nLC 1000 LC pump (Thermo Fisher Scientific, San Jose, CA). Peptides were separated using a 2 h gradient of 6 to 30% acetonitrile in 0.125% formic acid at a flow rate of 300 nL per min. Each analysis used a MultiNotch MS3-based TMT method. Mass spectra were processed using a Sequest-based in-house software pipeline. Data were searched using the human Uniprot database (April 2014) concatenated with common contaminants, and filtered to a final protein-level false discovery rate of 1%. The proteins were quantified by summing TMT reporter ion counts across all peptide-spectral matches using in-house software. For protein quantitation, reverse and contaminant proteins were removed, then each reporter ion channel was summed across all quantified proteins and normalized assuming equal protein loading across all samples^21^. Gene Ontology Cellular Compartment terms were added from www.uniprot.org and *p*-values determined using a two-tailed *t*-test adjusted with the Benjamini–Hochberg method using Perseus version 1.4.1.3^39^.

### Bioinformatic methods

The analysis of geographic distribution of coding variants in *ABCB6* was performed as previously described^15^, with some modifications. Genomic data were obtained from the Exome Aggregation Consortium (ExAC) database^27^, where data are stratified for different human populations based on geographic location. Populations were binned into groups with high or low malaria risk as determined based on the DALY (disability-adjusted life year per 100,000 population) values for malaria from the World Health Organization, with high exposure ≥10. For each coding variant, allele count and allele number were summed and Fisher’s exact test was used to determine the statistical significance for variant occurrence between the high and low malaria exposure groups, with the null hypothesis that the of variant occurrence in the two groups is the same.

### Data availability

The proteomics data generated during the current study are in the PRIDE repository (accession number PXD008752) and in Supplementary Table 1. All other relevant data are available from the authors upon request.

## Electronic supplementary material

Supplementary Information
