# Supplementary material for: Erythrocytes lacking the Langereis blood group protein ABCB6 are resistant to the malaria parasite Plasmodium falciparum
Source: Commun Biol. 2018 May 3;1:45. doi: 10.1038/s42003-018-0046-2 (PMC6123700; doi:10.1038/s42003-018-0046-2)
Supplement: Supplementary file 1 — Supplementary Information [file 42003_2018_46_MOESM1_ESM.pdf]

Supplementary Table 1: Proteomics data from five Lan null and five Lan-positive erythrocyte samples filtered by "plasma Membrane' GOCC annotation and > 1 peptide quantification

Uniprot	Description	Gene Symbol	Peptides	Ratio lan-null/Lan+	T-test B-H corrected	Lan-null_1 S:N	Lan-null_2 S:N	Lan-null_3 S:N	Lan-null_4 S:N	Lan-null_5 S:N	Lan+_1 S:N	Lan+_2 S:N	Lan+_3 S:N	Lan+_4 S:N	Lan+_5 S:N	Lan -ve sum S:N	Control sum S:N
Q9NP58	ATP-binding cassette sub-family B member 6, mitoc	ABCB6	15	0.07	0.00006	206	149	253	170	175	2303	2803	2354	3129	2581	952	13170
Q9UJU6-3	Isoform 3 of Drebrin-like protein	DBNL	4	0.29	0.00019	262	239	232	320	364	1133	864	1015	1065	883	1417	4960
P31641	Sodium- and chloride-dependent taurine transporte	SLC6A6	2	0.35	0.10395	174	172	176	483	453	1117	1359	747	654	275	1458	4152
P00749	Urokinase-type plasminogen activator	PLAU	6	0.37	0.13908	19	272	305	436	489	980	959	1206	977	14	1522	4136
Q6UVK1	Chondroitin sulfate proteoglycan 4	CSPG4	8	0.39	0.09243	270	487	494	532	484	1654	1557	1244	1038	357	2268	5850
Q9H2H9	Sodium-coupled neutral amino acid transporter 1	SLC38A1	3	0.39	0.09243	42	41	47	117	104	248	234	176	176	67	351	900
Q9UGT4	Sushi domain-containing protein 2	SUSD2	7	0.40	0.38253	1129	63	86	90	66	857	968	99	96	1597	1433	3617
P50570-4	Isoform 4 of Dynamin-2	DNM2	2	0.42	0.00038	88	91	59	55	51	157	157	171	172	166	344	822
Q96QD8	Sodium-coupled neutral amino acid transporter 2	SLC38A2	2	0.43	0.15029	24	26	30	68	67	138	154	95	83	25	215	495
P11413-2	Isoform Long of Glucose-6-phosphate 1-dehydroge	G6PD	5	0.46	0.00587	276	250	270	333	418	871	626	647	659	598	1549	3402
O43194	G-protein coupled receptor 39	GPR39	2	0.46	0.10395	92	192	220	344	350	661	650	591	568	148	1198	2617
P21589	5'-nucleotidase	NT5E	6	0.46	0.15092	273	600	583	1185	1024	2068	2309	1560	1731	306	3664	7974
P30825	High affinity cationic amino acid transporter 1	SLC7A1	5	0.48	0.11166	419	650	558	1087	1032	1687	1959	1873	1826	423	3747	7769
P46459	Vesicle-fusing ATPase	NSF	5	0.50	0.01793	459	429	297	225	192	628	661	789	605	550	1602	3233
Q01650	Large neutral amino acids transporter small subuni	SLC7A5	2	0.50	0.00298	82	61	68	81	86	176	158	119	159	149	378	760
Q9UNN8	Endothelial protein C receptor	PROCR	3	0.50	0.03686	186	248	279	348	280	621	651	564	550	308	1342	2695
P43121	Cell surface glycoprotein MUC18	MCAM	21	0.50	0.37706	277	2576	2763	1473	1456	1898	1928	6776	6128	328	8545	17058
P08237-3	Isoform 3 of 6-phosphofructokinase, muscle type	PFKM	7	0.50	0.10668	705	719	576	308	600	937	1047	1968	983	835	2907	5771
Q9Y653	G-protein coupled receptor 56	GPR56	3	0.51	0.29428	78	105	124	143	144	414	422	139	100	94	593	1168
P56199	Integrin alpha-1	ITGA1	3	0.51	0.11166	93	165	180	300	277	482	462	491	426	134	1015	1996
P37840	Alpha-synuclein	SNCA	6	0.51	0.01793	422	514	380	594	465	1211	889	751	781	1010	2375	4642
P11279	Lysosome-associated membrane glycoprotein 1	LAMP1	4	0.51	0.06038	195	201	138	276	418	505	301	587	512	495	1229	2400
P50281	Matrix metalloproteinase-14	MMP14	4	0.52	0.15565	144	401	440	434	396	783	890	893	787	138	1815	3490
Q658P3-2	Isoform 2 of Metalloreductase STEAP3	STEAP3	4	0.52	0.09243	474	404	440	524	541	1144	1145	960	897	393	2383	4540
P36269-3	Isoform 3 of Gamma-glutamyltransferase 5	GGT5	3	0.52	0.45380	87	326	339	277	288	950	1122	172	141	122	1316	2508
Q9UPY5	Cystine/glutamate transporter	SLC7A11	2	0.53	0.31728	303	284	318	1956	2225	2642	3030	1981	1690	314	5087	9658
Q03405	Urokinase plasminogen activator surface receptor	PLAUR	4	0.53	0.15618	51	86	84	122	133	184	219	237	220	41	476	899
Q00610	Clathrin heavy chain 1	CLTC	3	0.53	0.05861	151	54	68	73	107	166	142	195	137	209	453	848
P13639	Elongation factor 2	EEF2	2	0.54	0.05581	118	95	66	55	89	119	154	207	124	184	424	788
P00533	Epidermal growth factor receptor	EGFR	33	0.54	0.05838	5126	3179	3618	6664	6761	11319	11280	10065	7961	6402	25348	47027
P01130	Low-density lipoprotein receptor	LDLR	4	0.55	0.23459	107	557	599	653	663	912	949	1442	1261	153	2579	4717
P17813	Endoglin	ENG	11	0.55	0.10395	804	707	535	1367	1062	1550	1489	2177	1980	899	4475	8095
P30530	Tyrosine-protein kinase receptor UFO	AXL	4	0.55	0.43403	54	285	330	532	554	1239	1163	362	322	84	1754	3171
Q92692-2	Isoform Alpha of Poliovirus receptor-related protei	PVRL2	3	0.55	0.23448	50	197	208	211	214	300	314	506	403	61	879	1585
P29317	Ephrin type-A receptor 2	EPHA2	18	0.56	0.23448	684	1560	1868	2083	2287	3850	3784	4709	1909	920	8481	15172
Q96AP7	Endothelial cell-selective adhesion molecule	ESAM	2	0.56	0.25332	40	119	133	146	139	326	310	172	153	61	576	1022
Q04656-3	Isoform 2 of Copper-transporting ATPase 1	ATP7A	20	0.57	0.05962	2877	2495	2115	1317	1274	2698	3680	4548	3328	3429	10077	17683
P05556	Integrin beta-1	ITGB1	46	0.57	0.16782	1413	3118	3337	4593	4287	7853	7603	6770	5136	2023	16749	29385
Q14542	Equilibrative nucleoside transporter 2	SLC29A2	2	0.57	0.10395	38	61	54	45	41	90	103	61	116	49	239	420
P02786	Transferrin receptor protein 1	TFRC	31	0.57	0.04598	3732	1989	2295	2499	2441	5380	5380	4659	3345	3879	12956	22643
Q6YHK3	CD109 antigen	CD109	3	0.57	0.62087	405	28	24	28	23	50	61	154	169	452	508	886
Q15043	Zinc transporter ZIP14	SLC39A14	2	0.57	0.21842	109	120	125	332	299	505	455	310	289	158	984	1716
P08195-4	Isoform 4 of 4F2 cell-surface antigen heavy chain	SLC3A2	36	0.58	0.00778	3253	2598	2714	3859	3412	6122	6200	4782	5578	4820	15836	27501
O15431	High affinity copper uptake protein 1	SLC31A1	3	0.58	0.00298	112	126	150	177	161	240	272	259	260	224	725	1255
Q15758	Neutral amino acid transporter B(0)	SLC1A5	9	0.58	0.16633	258	456	483	781	785	974	1033	1298	1090	359	2763	4755
Q9NPQ8	Synembryn-A	RIC8A	3	0.59	0.10395	243	202	193	110	197	313	286	465	339	212	945	1615
Q09666	Neuroblast differentiation-associated protein AHN/	AHNAK	2	0.59	0.16343	47	38	45	76	84	133	142	84	68	66	290	492
Q92859	Neogenin	NEO1	2	0.59	0.10395	197	109	103	265	266	417	356	264	254	302	941	1593
P78536	Disintegrin and metalloproteinase domain-containi	ADAM17	6	0.59	0.09719	221	266	275	274	259	565	575	421	362	265	1295	2188
P26006-1	Isoform 2 of Integrin alpha-3	ITGA3	28	0.59	0.29428	999	2169	2368	3275	3273	6338	6459	3407	3000	1166	12085	20369
P09543	2',3'-cyclic-nucleotide 3'-phosphodiesterase	CNP	14	0.59	0.03112	621	741	547	306	505	936	904	1042	950	753	2720	4584
P04792	Heat shock protein beta-1	HSPB1	2	0.60	0.18583	44	68	38	34	75	73	95	64	59	144	259	434
P28827-2	Isoform 2 of Receptor-type tyrosine-protein phospl	PTPRM	8	0.60	0.26206	189	699	822	877	847	1804	1597	1000	818	466	3435	5685
P34910	Protein EVI2B	EVI2B	7	0.61	0.46577	110	242	245	944	920	1407	1355	723	461	103	2461	4050
O60266	Adenylate cyclase type 3	ADCY3	2	0.61	0.12247	132	123	123	202	197	353	338	211	195	183	779	1281
P30480	HLA class I histocompatibility antigen, B-42 alpha cl	HLA-B	2	0.61	0.33482	60	703	887	789	848	1195	1593	1399	1131	64	3286	5382
Q14126	Desmoglein-2	DSG2	2	0.61	0.13624	48	74	103	121	104	142	138	185	190	78	450	733
O15031	Plexin-B2	PLXNB2	11	0.61	0.11892	690	626	691	972	969	1804	1668	1043	985	930	3949	6430
Q13740	CD166 antigen	ALCAM	26	0.62	0.25332	1654	2753	3177	4293	3987	7334	7970	4445	3628	2314	15865	25691
Q14118	Dystroglycan	DAG1	10	0.63	0.15504	922	980	966	1253	1299	2410	2354	1422	1142	1257	5420	8586
P17301	Integrin alpha-2	ITGA2	29	0.63	0.23581	1068	3406	3557	3566	3661	5507	5334	6241	5661	1323	15257	24065
P16144	Integrin beta-4	ITGB4	18	0.63	0.32335	2489	2363	2789	2133	2398	6395	5959	1816	1728	3294	12171	19192
O00220	Tumor necrosis factor receptor superfamily membe	TNFRSF10A	2	0.64	0.35676	40	337	357	410	449	595	584	685	609	34	1594	2506
P04626	Receptor tyrosine-protein kinase erbB-2	ERBB2	14	0.64	0.69674	4385	308	313	703	697	1648	2144	320	311	5613	6406	10036
Q8NG11	Tetraspanin-14	TSPAN14	3	0.64	0.23304	68	103	112	171	170	261	273	180	169	92	625	975
P32004-2	Isoform 2 of Neural cell adhesion molecule L1	L1CAM	12	0.64	0.11892	622	905	954	1199	1158	1920	1657	1710	1436	809	4839	7532
O14672	Disintegrin and metalloproteinase domain-containi	ADAM10	4	0.64	0.10395	284	304	306	424	421	656	718	505	459	367	1738	2704
P23470	Receptor-type tyrosine-protein phosphatase gamm	PTPRG	4	0.64	0.29853	255	449	455	629	690	1163	1184	564	570	371	2478	3852
P07355-2	Isoform 2 of Annexin A2	ANXA2	8	0.65	0.17335	600	463	420	476	513	678	634	1259	712	537	2473	3820
P63010-2	Isoform 2 of AP-2 complex subunit beta	AP2B1	2	0.65	0.01793	159	118	159	160	187	272	221	208	233	267	784	1201
Q8WTV0	Scavenger receptor class B member 1	SCARB1	3	0.66	0.49200	1698	356	359	483	513	678	797	965	674	2085	3407	5199
P09758	Tumor-associated calcium signal transducer 2	TACSTD2	5	0.66	0.10395	269	200	214	172	159	230	235	390	348	342	1014	1545
P21333	Filamin-A	FLNA	4	0.66	0.02153	206	135	133	185	187	247	236	289	274	233	845	1278
Q9H5V8	CUB domain-containing protein 1	CDCP1	7	0.67	0.31060	272	849	937	878	931	1644	1502	1304	1049	310	3867	5809
Q9P0L0-2	Isoform 2 of Vesicle-associated membrane protein-	VAPA	2	0.67	0.23445	127	73	75	69	77	108	105	87	125	207	421	632
O14786	Neuropilin-1	NRP1	11	0.67	0.36514	224	678	708	1000	936	1557	1449	1098	955	235	3546	5295
Q96J84	Kin of IRRE-like protein 1	KIRREL	2	0.67	0.15029	46	92	76	81	86	144	117	137	109	61	381	568
Q92692	Poliovirus receptor-related protein 2	PVRL2	3	0.68	0.23448	56	138	136	122	126	177	180	239	184	73	579	853
Q96PD2-2	Isoform 2 of Discoidin, CUB and LCCL domain-conta	DCBLD2	4	0.68	0.23448	82	201	227	184	190	319	311	284	287	101	884	1302
Q9UIQ6	Leucyl-cystinyl aminopeptidase	LNPEP	3	0.68	0.11166	130	110	110	161	147	248	216	213	172	119	658	967
P08648	Integrin alpha-5	ITGA5	6	0.68	0.16633	317	445	499	681	616	906	887	816	725	406	2557	3740
P23229	Integrin alpha-6	ITGA6	31	0.69	0.17583	1992	2792	2977	4028	4038	4601	4276	6152	5382	2639	15826	23050
P15529-2	Isoform B of Membrane cofactor protein	CD46	5	0.69	0.05861	965	709	794	611	580	912	1015	1148	975	1267	3660	5317
O95297	Myelin protein zero-like protein 1	MPZL1	5	0.69	0.10395	154	232	240	219	219	275	260	386	373	244	1063	1538
Q9NPH3	Interleukin-1 receptor accessory protein	IL1RAP	2	0.70	0.19397	67	101	102	62	67	155	150	93	89	87	399	573
P18827	Syndecan-1	SDC1	2	0.70	0.74027	202	24	26	41	37	55	53	48	47	269	331	471
P58335	Anthrax toxin receptor 2	ANTXR2	4	0.71	0.45380	43	411	434	382	416	664	642	518	489	67	1685	2380
P04040	Catalase	CAT	18	0.71	0.16782	1608	1146	1452	1700	1816	1986	1679	3010	1576	2572	7722	10823
Q9Y289	Sodium-dependent multivitamin transporter	SLC5A6	3	0.72	0.10395	210	160	120	168	168	254	235	236	157	272	826	1154
P29323	Ephrin type-B receptor 2	EPHB2	10	0.72	0.33482	790	1704	1820	1814	1950	3009	3117	2306	2011	821	8077	11263
Q13443	Disintegrin and metalloproteinase domain-containi	ADAM9	2	0.72	0.10395	144	90	96	120	109	170	189	130	130	156	560	775
O00592	Podocalyxin	PODXL	2	0.73	0.46442	51	144	175	217	202	295	306	232	209	38	790	1079
P36507	Dual specificity mitogen-activated protein kinase ki	MAP2K2	3	0.73	0.15139	199	247	154	137	157	165	239	255	290	270	894	1220
P18564	Integrin beta-6	ITGB6	2	0.74	0.88347	698	15	17	4	4	4	15	16	37	930	739	1002
Q14517	Protocadherin Fat 1	FAT1	3	0.74	0.52161	37	94	89	110	113	208	186	91	76	41	443	601
Q9Y6M7-13	Isoform 13 of Sodium bicarbonate cotransporter 3	SLC4A7	2	0.74	0.57992	28	143	183	316	309	433	363	265	215	46	979	1322
P06756	Integrin alpha-V	ITGAV	16	0.75	0.59094	3056	1021	1111	1094	1020	1435	1450	1600	1365	3911	7301	9761
P19634	Sodium/hydrogen exchanger 1	SLC9A1	7	0.75	0.21710	510	369	393	353	325	502	476	445	417	764	1950	2604
P54709	Sodium/potassium-transporting ATPase subunit be	ATP1B3	14	0.75	0.17583	1322	2003	1650	2111	1701	2846	2932	2243	2113	1572	8786	11706
Q13308-6	Isoform 6 of Inactive tyrosine-protein kinase 7	PTK7	20	0.76	0.23448	1016	1634	1783	2130	2075	2514	2315	2809	2428	1360	8638	11426
Q9Y5Y0	Feline leukemia virus subgroup C receptor-related	FLVCR1	2	0.76	0.16721	104	51	62	59	74	106	95	74	90	97	349	462
P04920	Anion exchange protein 2	SLC4A2	8	0.76	0.84882	587	355	639	3929	426	459	471	372	503	6040	5936	7844
P55010	Eukaryotic translation initiation factor 5	EIF5	2	0.76	0.08166	380	320	318	253	268	405	385	365	390	485	1539	2030
P27105	Erythrocyte band 7 integral membrane protein	STOM	13	0.76	0.10395	1557	1282	1230	1034	1321	1417	2052	1386	1864	1747	6423	8466
P11021	78 kDa glucose-regulated protein	HSPA5	6	0.76	0.10395	197	345	322	247	275	367	350	440	320	349	1386	1826
Q95604	HLA class I histocompatibility antigen, Cw-17 alpha	HLA-C	3	0.77	0.49474	47	224	191	186	183	268	270	285	204	55	830	1082
O75369-8	Isoform 8 of Filamin-B	FLNB	2	0.78	0.17583	118	113	110	156	135	205	188	121	138	162	632	813
P42892	Endothelin-converting enzyme 1	ECE1	2	0.78	0.25332	282	304	325	384	382	566	531	362	293	389	1678	2141
P27701	CD82 antigen	CD82	2	0.79	0.45380	114	185	166	119	74	179	222	110	99	228	659	838
Q13641	Trophoblast glycoprotein	TPBG	3	0.79	0.01793	178	198	192	179	173	217	214	257	225	251	919	1164
P55011	Solute carrier family 12 member 2	SLC12A2	7	0.79	0.45380	496	637	631	1061	1014	1402	1276	839	746	597	3840	4861
P48509	CD151 antigen	CD151	2	0.79	0.40405	37	73	85	83	84	96	79	129	105	50	363	459
Q9Y4D1	Disheveled-associated activator of morphogenesis	DAAM1	2	0.79	0.24663	115	111	127	54	122	147	161	110	118	130	529	666

Q13530	Serine incorporator 3	SERINC3	4	0.80	0.28674	155	318	227	163	262	327	316	238	227	302	1125	1410
Q4KMQ2-2	Isoform 2 of Anoctamin-6	ANO6	4	0.80	0.10395	352	338	335	418	313	443	517	478	390	368	1755	2196
Q9Y6N7-2	Isoform 2 of Roundabout homolog 1	ROBO1	3	0.80	0.29428	152	138	183	236	235	294	275	213	226	167	944	1175
P15144	Aminopeptidase N	ANPEP	9	0.80	0.91393	4231	129	120	174	181	119	148	174	224	5353	4835	6018
P38606	V-type proton ATPase catalytic subunit A	ATP6V1A	2	0.81	0.19959	230	244	230	137	236	205	278	259	309	280	1077	1331
P48960	CD97 antigen	CD97	14	0.81	0.64527	425	2928	3283	2815	2981	3160	3393	4516	3826	459	12431	15354
P49327	Fatty acid synthase	FASN	4	0.81	0.25655	271	182	200	148	232	246	292	201	226	307	1033	1272
Q8IUW5	RELT-like protein 1	RELL1	7	0.81	0.37706	1692	2496	1620	910	1053	1736	1867	1983	2006	1969	7770	9561
P04406	Glyceraldehyde-3-phosphate dehydrogenase	GAPDH	17	0.83	0.25786	1258	1902	1714	1223	1593	1494	1672	2141	1718	2294	7689	9319
P01892	HLA class I histocompatibility antigen, A-2 alpha cha	HLA-A	4	0.83	0.65614	63	345	366	302	303	442	418	403	337	66	1379	1666
P11142	Heat shock cognate 71 kDa protein	HSPA8	18	0.83	0.10395	1363	1281	1215	1241	1409	1648	1571	1368	1378	1861	6509	7827
P01903	HLA class II histocompatibility antigen, DR alpha cha	HLA-DRA	2	0.84	0.77842	56	86	89	30	41	42	25	142	122	30	302	362
P54760	Ephrin type-B receptor 4	EPHB4	7	0.84	0.57644	370	580	347	325	710	691	330	421	648	672	2333	2762
P18433	Receptor-type tyrosine-protein phosphatase alpha	PTPRA	24	0.85	0.23448	1889	2567	2202	2048	1601	2524	2332	2197	2209	2917	10307	12179
Q9H7F0	Probable cation-transporting ATPase 13A3	ATP13A3	4	0.85	0.29428	241	176	199	184	183	251	268	268	176	192	984	1155
P53794	Sodium/myo-inositol cotransporter	SLC5A3	2	0.86	0.43403	121	75	74	67	76	102	107	86	107	75	413	478
Q9Y6M7	Sodium bicarbonate cotransporter 3	SLC4A7	3	0.87	0.75419	97	325	335	479	503	610	504	403	394	95	1739	2007
P35579	Myosin-9	MYH9	6	0.87	0.60949	428	216	225	222	209	292	267	273	241	426	1300	1499
P16070	CD44 antigen	CD44	73	0.88	0.34915	11530	13523	12328	15549	13689	12476	12689	17911	18141	14751	66619	75968
P13746-2	Isoform 2 of HLA class I histocompatibility antigen,	HLA-A	3	0.88	0.61697	123	313	278	273	229	272	306	351	287	166	1217	1381
Q9UHW9	Solute carrier family 12 member 6	SLC12A6	15	0.88	0.73534	3059	2430	1438	2085	1457	1379	2365	3848	2579	1673	10468	11845
P15151	Poliovirus receptor	PVR	7	0.89	0.46112	638	699	885	652	612	739	722	1039	801	627	3486	3927
Q9NQ25	SLAM family member 7	SLAMF7	2	0.89	0.82591	20	347	382	422	392	491	488	412	342	26	1562	1760
P01024	Complement C3	C3	16	0.89	0.82591	1647	1125	2365	1513	2515	4330	1082	1244	1431	2210	9165	10296
O15439	Multidrug resistance-associated protein 4	ABCC4	55	0.90	0.51242	9475	9500	7018	6797	6181	6605	9823	7801	10332	8883	38972	43443
P54578	Ubiquitin carboxyl-terminal hydrolase 14	USP14	4	0.90	0.23581	385	332	434	369	420	411	451	408	486	397	1940	2153
Q8TCZ2-5	Isoform 5 of CD99 antigen-like protein 2	CD99L2	7	0.90	0.83381	98	594	592	847	846	977	907	679	573	166	2977	3302
P53985	Monocarboxylate transporter 1	SLC16A1	9	0.90	0.83381	675	1307	440	983	303	750	579	829	552	1388	3707	4098
P13726	Tissue factor	F3	7	0.91	0.83963	123	440	490	606	585	445	442	771	705	112	2245	2475
P08575	Receptor-type tyrosine-protein phosphatase C	PTPRC	27	0.91	0.88116	5756	2098	4379	1804	1446	3398	2194	1902	2210	7347	15482	17051
Q7Z4W1	L-xylulose reductase	DCXR	2	0.91	0.46112	84	71	71	77	85	97	83	104	73	69	388	426
P17931	Galectin-3	LGALS3	10	0.92	0.69065	1230	1754	1178	1131	1139	1200	1038	1265	1725	1767	6432	6995
Q8N370-3	Isoform 3 of Large neutral amino acids transporter	SLC43A2	11	0.93	0.77842	2545	2505	2532	1130	2066	2311	1582	2109	2344	3267	10777	11613
P53396	ATP-citrate synthase	ACLY	10	0.95	0.79915	508	465	508	734	919	726	671	614	628	676	3135	3315
P01857	Ig gamma-1 chain C region	IGHG1	4	0.95	0.82440	358	285	332	317	281	481	301	249	264	356	1572	1652
P05362	Intercellular adhesion molecule 1	ICAM1	11	0.95	0.89074	372	1069	1165	698	775	838	803	1163	974	496	4080	4274
Q9UIW2	Plexin-A1	PLXNA1	19	0.96	0.77037	1194	1497	1653	1807	1549	1973	1691	1428	1360	1585	7701	8037
P49368	T-complex protein 1 subunit gamma	CCT3	10	0.96	0.84882	682	625	859	422	701	654	551	844	600	774	3290	3423
P30043	Flavin reductase (NADPH)	BLVRB	6	0.96	0.84882	1064	764	1182	1540	1460	1167	1094	1046	1467	1476	6009	6250
P02730	Band 3 anion transport protein	SLC4A1	619	0.97	0.62386	78261	85840	83090	83901	95472	89448	89544	82929	97908	81271	426564	441101
Q9Y6M5	Zinc transporter 1	SLC30A1	26	0.98	0.90095	6080	4428	3917	4361	3732	4002	4101	5214	4844	4835	22519	22996
P49281-3	Isoform 3 of Natural resistance-associated macroph	SLC11A2	5	0.98	0.95279	328	601	329	563	346	443	329	706	506	227	2166	2212
Q12913	Receptor-type tyrosine-protein phosphatase eta	PTPRJ	4	0.98	0.94202	132	274	296	327	328	324	307	323	270	161	1358	1385
P05026	Sodium/potassium-transporting ATPase subunit be	ATP1B1	25	0.98	0.93796	1969	4147	4505	3951	4097	4327	4453	4311	3465	2486	18671	19041
P11277-2	Isoform 2 of Spectrin beta chain, erythrocytic	SPTB	3	0.99	0.94202	170	152	164	220	183	166	200	163	132	239	889	901
P51790	H(+)/Cl(-) exchange transporter 3	CLCN3	8	0.99	0.96675	656	504	450	474	319	326	424	759	581	341	2403	2431
P14672	Solute carrier family 2, facilitated glucose transport	SLC2A4	3	0.99	0.94526	277	335	367	377	313	262	312	404	326	382	1669	1685
P0C0L4	Complement C4-A	C4A	18	0.99	0.99603	1407	1423	2019	1680	7274	8782	816	1151	1452	1712	13802	13914
Q9NV96	Cell cycle control protein 50A	TMEM30A	13	0.99	0.96675	1614	1828	1707	1546	1305	1267	1748	1667	1910	1451	8000	8042
A1A5C7	Solute carrier family 22 member 23	SLC22A23	18	1.00	0.99603	2794	2277	2519	2401	1587	1872	2040	1887	2580	3209	11579	11587
P08581	Hepatocyte growth factor receptor	MET	5	1.00	0.99603	724	655	717	760	741	850	830	635	633	650	3597	3600
Q8IWA5	Choline transporter-like protein 2	SLC44A2	8	1.01	0.95417	839	1120	989	867	842	612	765	1083	1000	1150	4657	4610
Q8NB49	Probable phospholipid-transporting ATPase IG	ATP11C	4	1.01	0.93333	603	680	653	717	611	557	811	684	705	461	3263	3218
P07900-2	Isoform 2 of Heat shock protein HSP 90-alpha	HSP90AA1	11	1.02	0.93364	708	684	966	886	911	902	824	624	674	1066	4156	4089
P62834	Ras-related protein Rap-1A	RAP1A	11	1.02	0.86550	1075	1333	1202	926	1092	913	1220	1063	1260	1052	5629	5508
P25445	Tumor necrosis factor receptor superfamily membe	FAS	7	1.03	0.91617	2594	4023	3232	2215	1785	2525	2152	2584	2036	4089	13849	13386
Q92581-2	Isoform 2 of Sodium/hydrogen exchanger 6	SLC9A6	3	1.04	0.84882	275	223	178	203	158	152	195	215	230	210	1038	1003
P06733	Alpha-enolase	ENO1	8	1.05	0.69674	662	483	631	591	701	470	642	556	580	671	3067	2919
P43007	Neutral amino acid transporter A	SLC1A4	2	1.05	0.82325	206	195	145	305	231	169	227	215	244	170	1082	1026
P28907	ADP-ribosyl cyclase 1	CD38	16	1.05	0.74821	1557	1491	1423	1013	1166	1289	1043	1448	1051	1476	6651	6308
Q5T3F8	Transmembrane protein 63B	TMEM63B	7	1.06	0.81143	925	781	641	659	450	574	592	815	737	557	3456	3275
P24666	Low molecular weight phosphotyrosine protein pho	ACP1	8	1.07	0.60949	1340	1377	1040	984	987	925	1180	985	1188	1071	5728	5349
Q7KYR7	Butyrophilin subfamily 2 member A1	BTN2A1	4	1.08	0.66151	398	440	326	342	522	456	416	368	376	257	2028	1873
Q9UNQ0	ATP-binding cassette sub-family G member 2	ABCG2	10	1.09	0.82440	732	932	1585	1523	2409	1714	1029	1296	1143	1435	7182	6617
P0C0L5	Complement C4-B	C4B	4	1.09	0.92642	108	106	227	102	440	482	72	98	111	142	983	905
Q16563	Synaptophysin-like protein 1	SYPL1	6	1.09	0.75414	1026	1359	625	735	574	749	981	758	769	690	4319	3947
Q9Y5S1	Transient receptor potential cation channel subfam	TRPV2	2	1.09	0.70360	243	163	137	118	150	116	177	130	142	176	811	741
P18577	Blood group Rh(CE) polypeptide	RHCE	10	1.11	0.28203	1399	1329	1531	1649	1410	1212	1331	1093	1450	1501	7318	6587
Q92508	Piezo-type mechanosensitive ion channel compone	PIEZO1	41	1.12	0.50420	5043	4314	3720	6073	5801	3942	4477	4817	3582	5525	24951	22344
Q9NZW5	MAGUK p55 subfamily member 6	MPP6	2	1.12	0.59094	140	213	137	125	122	109	118	173	132	128	737	660
O15440	Multidrug resistance-associated protein 5	ABCC5	21	1.12	0.52161	2927	2794	1809	2250	1968	1550	2573	2306	2224	1814	11748	10467
P04156	Major prion protein	PRNP	2	1.12	0.47230	122	182	193	142	126	120	135	150	159	117	764	680
Q92542	Nicastrin	NCSTN	4	1.13	0.40920	355	419	307	349	257	291	241	291	344	329	1687	1497
P29972	Aquaporin-1	AQP1	3	1.13	0.46347	1349	1055	1011	1026	1108	810	900	784	1037	1376	5549	4907
Q15223	Poliovirus receptor-related protein 1	PVRL1	18	1.13	0.49200	2876	3636	2337	2977	2241	2018	2000	2717	2440	3238	14067	12414
P11171-2	Isoform 2 of Protein 4.1	EPB41	18	1.13	0.36514	2955	2159	2248	2450	2012	1868	2108	1775	2086	2593	11823	10431
P67775	Serine/threonine-protein phosphatase 2A catalytic	PPP2CA	2	1.13	0.38074	195	178	184	166	265	190	161	167	190	163	987	871
P05023	Sodium/potassium-transporting ATPase subunit alp	ATP1A1	30	1.14	0.23581	2473	3687	3171	3465	2949	2921	2807	2804	2526	2780	15744	13838
Q8IZY2	ATP-binding cassette sub-family A member 7	ABCA7	25	1.15	0.74020	2917	2479	1755	2169	1981	1188	1386	1622	1634	4038	11300	9867
P02549	Spectrin alpha chain, erythrocytic 1	SPTA1	7	1.16	0.43071	501	407	483	624	453	445	447	309	354	579	2468	2135
P19440	Gamma-glutamyltranspeptidase 1	GGT1	3	1.17	0.63972	366	510	512	254	249	218	222	371	310	500	1890	1621
P21980	Protein-glutamine gamma-glutamyltransferase 2	TGM2	15	1.17	0.37764	2101	1896	1630	1654	2178	2111	1622	1634	1769	967	9460	8103
P35613	Basigin	BSG	54	1.17	0.36514	7150	9202	6858	9375	5352	5747	5860	6149	6992	7723	37937	32471
P06703	Protein S100-A6	S100A6	5	1.17	0.25786	807	712	727	771	1072	677	788	608	699	728	4089	3500
Q93050-3	Isoform 3 of V-type proton ATPase 116 kDa subunit	ATP6V0A1	4	1.17	0.49927	337	247	202	217	241	185	186	331	205	156	1245	1062
P04899-4	Isoform sGi2 of Guanine nucleotide-binding protein	GNAI2	4	1.17	0.11166	184	248	254	236	240	199	203	215	189	186	1162	990
Q9Y666	Solute carrier family 12 member 7	SLC12A7	10	1.19	0.64527	1664	947	537	1236	637	649	808	1168	565	1041	5021	4231
P63000-2	Isoform B of Ras-related C3 botulinum toxin substra	RAC1	15	1.19	0.14802	1731	1741	1788	1937	2151	1417	1684	1905	1538	1330	9347	7874
O15438-4	Isoform 4 of Canalicular multispecific organic anion	ABCC3	3	1.19	0.03441	208	219	200	196	200	157	196	174	169	165	1022	861
Q9Y624	Junctional adhesion molecule A	F11R	20	1.20	0.31487	2654	2520	2319	2513	2040	1622	1217	2574	2221	2425	12047	10058
P16157-14	Isoform Er13 of Ankyrin-1	ANK1	128	1.20	0.23448	20313	19804	20549	26666	28655	21976	21859	18148	18865	15528	115986	96376
P11166	Solute carrier family 2, facilitated glucose transport	SLC2A1	172	1.21	0.09719	24553	22994	23306	24615	22917	15679	19351	18507	23647	20853	118386	98037
P07384	Calpain-1 catalytic subunit	CAPN1	22	1.21	0.11892	2054	1909	1879	1839	2361	1355	1751	1671	1979	1536	10042	8292
P00918	Carbonic anhydrase 2	CA2	13	1.21	0.51147	1360	1017	1814	854	902	726	722	913	1049	1487	5946	4896
P14415	Sodium/potassium-transporting ATPase subunit be	ATP1B2	10	1.22	0.31487	2411	2906	1856	2057	1780	1545	1343	1936	1775	2424	11009	9024
P01876	Ig alpha-1 chain C region	IGHA1	3	1.22	0.29428	265	241	191	250	271	277	137	199	155	230	1217	997
Q93070	Ecto-ADP-ribosyltransferase 4	ART4	13	1.22	0.43506	2504	3216	1672	1980	1333	1332	1959	1930	1755	1788	10704	8764
Q08722	Leukocyte surface antigen CD47	CD47	47	1.23	0.10395	6210	6662	5336	4862	4901	4593	4393	4179	4814	4829	27970	22808
Q9Y2G3	Probable phospholipid-transporting ATPase IF	ATP11B	2	1.24	0.10395	145	159	149	134	124	83	118	116	127	130	710	573
P16452-2	Isoform Long of Erythrocyte membrane protein ban	EPB42	47	1.25	0.11166	6020	5943	7191	6677	6410	4745	6827	5070	5010	4084	32241	25735
P98172	Ephrin-B1	EFNB1	12	1.26	0.41489	2035	1925	1557	1259	1188	1042	834	1195	1155	2116	7964	6342
O00186	Syntaxin-binding protein 3	STXBP3	5	1.27	0.23687	362	626	669	599	481	341	440	452	554	373	2736	2159
Q9NP72-2	Isoform 2 of Ras-related protein Rab-18	RAB18	3	1.27	0.16782	672	567	722	596	824	482	546	627	641	365	3381	2661
Q02094	Ammonium transporter Rh type A	RHAG	12	1.28	0.08864	3597	3124	3139	2851	2660	2116	2211	2202	2628	2860	15371	12018
P78310	Coxsackievirus and adenovirus receptor	CXADR	8	1.28	0.23459	1086	1011	916	1383	824	512	850	807	1076	829	5219	4073
Q96PL5	Erythroid membrane-associated protein	ERMAP	11	1.29	0.03119	2185	2242	2356	2181	2307	2048	1417	1904	1603	1794	11271	8765
P19256	Lymphocyte function-associated antigen 3	CD58	18	1.30	0.21710	2370	2487	1850	1782	1340	1098	1430	1550	1738	1759	9830	7574
P22413	Ectonucleotide pyrophosphatase/phosphodiesteras	ENPP1	13	1.30	0.69065	188	2298	2323	844	896	1682	1554	878	761	158	6550	5032
P13987	CD59 glycoprotein	CD59	17	1.32	0.28203	1570	2259	1614	1306	1197	825	1022	1270	1807	1082	7946	6006
P23634	Plasma membrane calcium-transporting ATPase 4	ATP2B4	12	1.32	0.05224	1669	1700	1559	2172	1749	1271	1495	1228	1425	1262	8849	6681
P01116-2	Isoform 2B of GTPase KRas	KRAS	2	1.33	0.03686	209	214	226	248	277	154	180	185	193	174	1174	885
O00299	Chloride intracellular channel protein 1	CLIC1	3	1.33	0.10668	386	413	305	421	315	262	224	331	340	228	1840	1385
P04632	Calpain small subunit 1	CAPNS1	2	1.34	0.15029	151	138	148	190	238	138	117	133	119	140	864	647
P02724	Glycophorin-A	GYPA	80	1.34	0.17615	14989	12480	10860	9807	9729	7611	7230	7051	8366	12813	57866	43072
P61006	Ras-related protein Rab-8A	RAB8A	2	1.35	0.04902	142	168	166	207	154	130	131	110	123	125	836	619
Q14773	Intercellular adhesion molecule 4	ICAM4	44	1.35	0.16782	7933	6727	7355	6103	6311	4489	3299	4457	5288	7959	34429	25493

O15554	Intermediate conductance calcium-activated potas:	KCNN4	9	1.35	0.01793	1582	1479	1287	1565	1543	1164	962	1065	1030	1296	7457	5517
P08174-7	Isoform 7 of Complement decay-accelerating factoı	CD55	42	1.36	0.25786	10854	12950	8046	7721	5577	4035	7066	6949	7809	7436	45147	33296
P41440	Folate transporter 1	SLC19A1	3	1.36	0.10395	228	235	158	264	224	141	181	135	170	187	1109	814
P61225	Ras-related protein Rap-2b	RAP2B	6	1.37	0.11166	714	681	670	487	586	378	426	390	444	659	3138	2296
P33527-9	Isoform 9 of Multidrug resistance-associated protei	ABCC1	30	1.39	0.08166	3705	3902	4408	5226	4861	3291	3987	3315	2845	2407	22102	15845
P20020	Plasma membrane calcium-transporting ATPase 1	ATP2B1	11	1.41	0.04468	1120	1091	991	1463	1080	730	825	782	878	851	5745	4066
P16671	Platelet glycoprotein 4	CD36	5	1.43	0.15618	451	535	317	427	256	251	256	239	278	367	1986	1391
P04921	Glycophorin-C	GYPC	67	1.48	0.04598	4972	4537	3855	3836	3502	2402	2590	2444	2933	3624	20702	13993
O60486	Plexin-C1	PLXNC1	3	1.49	0.32110	445	773	510	335	514	5	308	518	354	542	2576	1728
Q99808	Equilibrative nucleoside transporter 1	SLC29A1	35	1.49	0.10395	3991	5306	4274	4763	3429	2138	2271	2771	2857	4536	21763	14573
Q01973	Tyrosine-protein kinase transmembrane receptor R	ROR1	3	1.53	0.31419	68	341	313	455	453	226	190	288	268	90	1630	1062
P23276	Kell blood group glycoprotein	KEL	41	1.56	0.10395	4700	4961	4266	4706	3959	3770	754	3495	3577	2903	22592	14499
P22303-2	Isoform H of Acetylcholinesterase	ACHE	20	1.58	0.09243	3629	3736	2769	3414	2023	1377	2149	1769	2015	2549	15571	9859
Q08174-2	Isoform 2 of Protocadherin-1	PCDH1	4	1.59	0.43562	555	381	312	265	230	114	115	115	122	628	1743	1094
P51811	Membrane transport protein XK	XK	10	1.61	0.10395	1691	1615	1353	1828	1227	1297	243	1209	1081	976	7714	4805
O75387	Large neutral amino acids transporter small subuni	SLC43A1	7	1.62	0.12599	387	1004	1015	1059	743	448	492	539	459	665	4208	2603
Q8WUD1	Ras-related protein Rab-2B	RAB2B	2	1.66	0.01674	281	321	324	357	286	163	178	257	203	146	1569	948
P02760	Protein AMBP	AMBP	4	1.66	0.13816	431	387	444	530	391	187	200	188	197	541	2184	1313
P51148-2	Isoform 2 of Ras-related protein Rab-5C	RAB5C	4	1.68	0.01228	309	369	380	358	371	234	210	283	191	148	1787	1066
P00568	Adenylate kinase isoenzyme 1	AK1	4	1.85	0.10395	318	189	319	527	485	205	182	194	219	192	1837	991
Q00013	55 kDa erythrocyte membrane protein	MPP1	22	1.87	0.00019	3626	3425	3549	4213	3504	1961	1960	2007	1926	1939	18317	9793
Q13336-2	Isoform 2 of Urea transporter 1	SLC14A1	10	1.91	0.15504	1865	1419	1772	1556	1634	1174	1282	48	47	1765	8246	4316
O15162	Phospholipid scramblase 1	PLSCR1	2	1.92	0.10395	182	198	209	412	297	154	124	136	118	143	1298	675
P61106	Ras-related protein Rab-14	RAB14	2	1.95	0.00215	442	428	499	558	461	228	240	286	276	194	2388	1224
P17927	Complement receptor type 1	CR1	37	1.98	0.08864	6752	7894	5455	10091	4884	1764	3453	3903	3079	5494	35076	17694
P60953	Cell division control protein 42 homolog	CDC42	2	2.05	0.04598	250	169	272	333	380	130	166	142	125	123	1404	686
O75695	Protein XRP2	RP2	2	2.09	0.13999	162	167	183	429	209	92	100	100	151	107	1150	550
P50895	Basal cell adhesion molecule	BCAM	39	2.24	0.10395	8458	9444	8912	4477	3775	3536	1179	3046	2073	5799	35066	15633
Q9NRQ2	Phospholipid scramblase 4	PLSCR4	3	2.50	0.10395	197	180	179	489	297	113	112	87	88	138	1343	538
Q8IZF2	Probable G-protein coupled receptor 116	GPR116	4	2.55	0.22930	130	1956	2168	872	1028	260	241	943	853	120	6153	2417
P01008	Antithrombin-III	SERPINC1	3	2.59	0.09559	130	226	190	161	236	97	24	24	26	193	943	364
O75326	Semaphorin-7A	SEMA7A	15	2.65	0.10395	2554	3064	1546	1678	889	161	333	891	1025	1265	9730	3674
Q9NP59	Solute carrier family 40 member 1	SLC40A1	45	2.83	0.01359	8480	11959	9301	9327	7992	5129	1280	1993	2528	5723	47059	16653
P0CG48	Polyubiquitin-C	UBC	14	2.84	0.00298	2604	1753	1866	2264	2694	664	1017	652	787	820	11182	3939
O00421-2	Isoform 2 of C-C chemokine receptor-like 2	CCRL2	6	2.93	0.05447	592	1025	1051	700	705	209	191	135	127	729	4072	1392
P14209	CD99 antigen	CD99	5	5.50	0.02391	1612	474	1025	1500	1264	412	116	160	114	266	5876	1068

Supplementary Table 2: *ABCB6* coding region or splice variants enriched in geographic regions with low (A) or high (B) malaria exposure as percent of population, from the ExAC database

A)

ΔAA*		p.Ile797Val	p.Val780Ile	p.Val772Phe	p.Arg739Cys	p.Gly730Arg	c.106-5G>A	p.Ala681Thr	c.1863+8G>C	p.Tyr599Cys	p.Gly588Ser
rs[#]					rs141840760	rs146941118		rs142421126			rs145526996
Predicted Effect		missense	missense	missense	missense	missense	splice region	missense	splice region	missense	missense
Pops[&]	Exp[%]										
Afr	high	0	0	0	0	0	0	0	0	0	0.14
S.A.	high	0	0.01	0	0.01	0.01	0.02	0.01	0.33	0	0.1
E.A.	low	0.23	0	0	0	0	0.5	0	0	0.28	0
Fin	low	0	0	0	0	0	0	0.06	0	0	0.39
Euro	low	0	0.06	0.02	0.05	0.01	0.01	0.04	0.02	0	0.71
Lat	low	0	0.03	0	0.08	0.15	0.01	0	0	0	0.28
**P-value****		**0.012426696**	**0.00015518**	**0.031135135**	**0.000585064**	**0.018310047**	**0.003373824**	**0.004994011**	**6.95E-25**	**0.005011035**	**3.54E-28**

ΔAA*		p.Ala492Thr	p.Val454Ala	p.Arg365His	p.Arg276Gln[δ]	p.Arg276Trp	p.Arg247Cys	p.Glu220Val	p.Arg192Gln	p.Arg192Trp[δ]
rs[#]		rs147445258	rs61733629		rs200125320	rs57467915	rs190528998	rs142256852	rs150221689	rs149202834
Predicted Effect		missense	missense	missense	missense	missense	missense	missense	missense	missense
Pops[&]	Exp[%]									
Afr	high	0.19	0.04	0	0	0.39	0	0	0.05	0.01
S.A.	high	0.02	0.01	0	0.01	0.38	0	0	0.07	0.03
E.A.	low	0	0.05	0	0	0.07	0	0	0	0
Fin	low	0.97	0	0	0.45	6.53	0	0.03	0.15	0.18
Euro	low	0.95	0	0.02	0.1	2.96	0.01	0.02	0.51	0.3
Lat	low	1.16	4.69	0.16	0	1.05	6.5	0	0.4	0.04
**P-value****		**1.31E-61**	**1.42E-52**	**0.002800279**	**0.000507627**	**1.69E-68**	**1.26E-67**	**0.019287316**	**6.51E-26**	**6.08E-17**

B)

ΔAA*		p.Thr778Ile	p.Gly715Glu	p.Arg685His	p.Arg685Cys	p.Asp682Asn	p.Asn677Asp	p.Ala660Val	p.Ala660Asp	p.Arg648Gln[δ]	c.1579-7G>A	c.1578+5G>C
rs[#]		rs143621292			rs139523052	rs144295428		rs140239756	rs140239756	rs13402964		
Predicted Effect		missense	missense	missense	missense	missense	missense	missense	missense	missense	splice region	splice region
Pops[&]	Exp[%]											
Afr	high	0.1	0	0	0.13	0.03	0	0.03	0.04	0.64	0	0.05
S.A.	high	0	0.02	0.02	0	0.01	0.05	0	0	0	0.1	0
E.A.	low	0	0	0.02	0	0	0	0	0	0.01	0	0
Fin	low	0	0	0	0	0	0	0	0	0	0	0
Euro	low	0	0	0	0	0	0	0	0	0.01	0.01	0
Lat	low	0	0	0	0.03	0	0	0	0	0.05	0.05	0
**P-value****		**3.10E-07**	**0.011221114**	**0.02526153**	**9.28E-06**	**0.010227726**	**6.19E-06**	**0.011134939**	**0.002486329**	**1.31E-30**	**8.45E-05**	**0.000518769**

ΔAA*		p.Thr521Ser	p.Gly512Ser	p.Ala511Thr	p.Gln499Leu	p.Val495Leu	p.Arg475Cys	p.Ser470Asn	p.Glu469Gln	p.Leu425Val	c.1154+2T>C	p.Arg371Gln
rs[#]		rs149363094		rs140089441			rs140244896		rs111677240	rs111852229		
Predicted Effect		missense	missense	missense	missense	missense	missense	missense	missense	missense	splice donor	missense
Pops[&]	Exp[%]											
Afr	high	1.54	0.01	0.16	0.04	0	0.04	0	0	0.44	0	0
S.A.	high	0.12	0.01	0.01	0	0.04	0	0.02	0.04	0.01	0.04	0.14
E.A.	low	0	0	0	0	0	0.01	0	0	0	0	0
Fin	low	0.05	0	0	0	0	0	0	0	0	0	0
Euro	low	0.38	0	0	0	0	0	0	0	0	0	0.01
Lat	low	0.28	0	0	0	0.01	0.01	0	0	0.03	0	0
**P-value****		**7.63E-14**	**0.035997645**	**7.67E-10**	**0.00241702**	**0.000676604**	**0.048561577**	**0.011148228**	**2.78E-05**	**2.44E-26**	**0.004688397**	**9.16E-09**

ΔAA*		p.Glu355Lys	p.Phe332Leu	p.Val306Ile	p.Leu302Val	p.Arg231Gln	p.Ala164Thr	p.Ala57Thr	p.Phe29del[δ]	p.Pro13Leu	p.Cys8SerfsTe	p.Val2Met
rs[#]			rs138423826		rs113159519		rs143052685					
Predicted Effect		missense	missense	missense	missense	missense	missense	missense	inframe deleti	missense	frameshift	missense
Pops[&]	Exp[%]											
Afr	high	0.06	0.17	0	0.11	0	0.13	0	0	0	0	0.04
S.A.	high	0	0	0.26	0	0.05	0	0.08	0.03	0.02	0.02	0
E.A.	low	0	0	0	0	0	0	0	0.02	0	0	0
Fin	low	0	0	0	0	0	0	0	0	0	0	0
Euro	low	0	0	0	0	0	0.01	0	0	0	0	0
Lat	low	0	0.02	0	0.01	0	0.04	0.01	0	0	0	0
**P-value****		**0.018214486**	**9.74E-07**	**3.73E-27**	**6.74E-07**	**8.38E-05**	**0.001156826**	**4.64E-07**	**0.030898784**	**0.040922968**	**0.037774826**	**0.006369975**

*, Amino acid change and location

#, Reference SNP cluster ID (NCBI)

&, Populations: Africans; S.A, South Asians; E.A., East Asians; Fin, Finnish; Euro, non-Finnish Europeans; Lat, Latinos.

%, Exposure of current or ancestral popluation to malaria based on World Health Organization DALY.

δ, Variant previously reported for Lan-null blood type

**, Fisher's exact test comparing populations binned for high or low malaria exposure.
